# HIV incidence declines in a rural South African population: a G-imputation approach for inference

**DOI:** 10.1186/s12889-020-09193-4

**Published:** 2020-08-06

**Authors:** Alain Vandormael, Diego Cuadros, Adrian Dobra, Till Bärnighausen, Frank Tanser

**Affiliations:** 1 Africa Health Research Institute (AHRI), Durban, South Africa; 2 Heidelberg Institute of Global Health, University of Heidelberg, 130.3 Im Neuenheimer Feld, Heidelberg, 69115 Germany; 3 KwaZulu-Natal Research Innovation and Sequencing Platform (KRISP), University of KwaZulu-Natal (UKZN), Durban, South Africa; 4 Department of Geography and Geographic Information Science, University of Cincinnati, Cincinnati, USA; 5 Department of Statistics, Center for Statistics and the Social Sciences, and Center for Studies in Demography and Ecology, University of Washington, Seattle, USA; 6 Department of Global Health and Population, Harvard T.H. Chan School of Public Health, Boston, USA; 7 Lincoln International Institute for Rural Health, University of Lincoln, Lincoln, UK; 8 School of Nursing and Public Health, UKZN, Durban, South Africa; 9 Centre for the AIDS Programme of Research in South Africa (CAPRISA), Durban, South Africa

**Keywords:** HIV, Incidence, G-imputation, Random-point, Estimation, South Africa

## Abstract

**Background:**

Ad hoc assumptions about the unobserved infection event, which is known only to occur between the latest-negative and earliest-positive test dates, can lead to biased HIV incidence rate estimates. Using a G-imputation approach, we infer the infection dates from covariate data to estimate the HIV incidence rate in a hyper-endemic South African setting.

**Methods:**

A large demographic surveillance system has annually tested a cohort of HIV-uninfected participants living in the KwaZulu-Natal province. Using this data, we estimated a cumulative baseline hazard function and the effects of time-dependent covariates on the interval censored infection dates. For each HIV-positive participant in the cohort, we derived a cumulative distribution function and sampled multiple infection dates conditional on the unique covariate values. We right censored the data at the imputed dates, calculated the annual HIV incidence rate per 100 person-years, and used Rubin’s rules to obtain the 95% confidence intervals.

**Results:**

A total of 20,011 uninfected individuals with a repeat HIV test participated in the incidence cohort between 2005 and 2018. We observed 2,603 infections per 58,769 person-years of follow-up among women and 845 infections per 41,178 person-years of follow-up among men. Conditional on age and circumcision status (men only), the female HIV incidence rate declined by 25%, from 5.0 to 3.7 infections per 100 person-years between 2014 and 2018. During this period, the HIV incidence rate among men declined from 2.1 to 1.1 infections per 100 person-years—a reduction of 49%. We observed similar reductions in male and female HIV incidence conditional on condom-use, marital status, urban residential status, migration history, and the HIV prevalence in the surrounding community.

**Conclusion:**

We have followed participants in one of the world’s largest and longest running HIV cohorts to estimate long-term trends in the population-wide incidence of infection. Using a G-imputation approach, we present further evidence for HIV incidence rate declines in this hyper-endemic South African setting.

## Background

There is an urgent need to assess the impact of treatment and prevention services on the population-wide incidence of HIV infection in sub-Saharan Africa [1]. One way to quantify the incidence rate is to repeatedly test a cohort of uninfected participants until they are HIV-positive [2]. However, population-based cohorts are logistically difficult and costly to maintain, with test dates typically scheduled on a periodic basis, say every six, twelve, or twenty-four months. This means that we rarely (if ever) observe the infection event, which is censored at some time-point between the latest HIV-negative and earliest HIV-positive test dates, otherwise known as the censoring interval. Furthermore, participants may miss their scheduled test dates, either due to testing fatigue, sickness, work commitments, or out-migration (among other reasons) [3–5], thus increasing the censored interval length and our uncertainty about the timing of the infection event [6].

Several ad-hoc imputation strategies have been adopted to address the interval censoring problem. The most popular of these is to assume that the infection event occurs at the mid-point of the censoring interval. However, our research shows that mid-point imputation can lead to severely biased annual incidence rates once the censoring interval extends across one or more years [6]. In a simulation analysis, we demonstrated that the single random-point method, when coupled with standard multiple imputation procedures, produced estimates (with appropriate standard errors) close to the true incidence rate [6]. Nevertheless, one limitation of the single random-point method is that it discards important auxiliary information about the timing of the infection event [7]. For example, we know that participants will have, on average, shorter times to infection if they are young and female (aged 15–24 years) or live in communities with a high prevalence of HIV [8, 9]. Such information, which is routinely collected by surveillance systems during HIV testing, could reduce uncertainty about the infection date and improve the accuracy of the incidence rate estimates.

To estimate the HIV incidence rate, we use a novel method called G-imputation to infer the unobserved infection dates from covariate data [10]. First, we leverage a G-transformation model to estimate the cumulative baseline hazard function and the effects of time-dependent covariates on the interval censored data [11]. Next, we derive a cumulative distribution function for each HIV-positive participant. From this distribution, we sample multiple infection dates conditional on the participant’s covariate values [10]. The data come from a large, population-based HIV surveillance system in the north-east region of South Africa. In this paper, we undertake a first empirical analysis of this data using the G-imputation approach, motivate our selection of covariates to infer the infection dates, and assess the impact of model selection on the incidence rate estimates. We further compare our G-imputation results with previously published estimates using single random-point imputation. This work continues our efforts to address the interval censoring problem and accurately estimate the incidence of HIV in a hyper-endemic South African setting.

## Methods

### Surveillance area

Since 2000, the Africa Health Research Institute (AHRI) has maintained a comprehensive, population-based surveillance system in the uMkanyakude district of the KwaZulu-Natal province. The surveillance system has been designed to capture the complex and dynamic demographic reality of its surrounding community [18]. With a size of 438 km^2^, the surveillance area is home to approximately 90,000 persons living in 11,000 households. Households are mostly scattered across the landscape, with several informal peri-urban settlements and a single urban township. People living in the surveillance area are generally poor and frequently migrate to urban or industrial areas for long periods of time [12]. The majority of the participants are Zulu-speaking (black) Africans.

### Household and HIV surveys

Two to three times yearly, trained field-workers visit all households in the surveillance area. Household attributes are recorded and a senior household member provides information on the birth, death, relationship, and migration history of household residents. Since 2004, an annual HIV survey has been nested in the household survey. Men between the ages of 15 and 54 years and women between the ages of 15 and 49 years are eligible for HIV testing. After obtaining written consent, field workers administer individual questionnaires in private and extract blood according to the *UNAIDS and WHO Guidelines for Using HIV Testing Technologies in Surveillance* [13]. The Household and HIV surveys, and the respective participation rates, are described in greater detail elsewhere [17].

### Statistical methods

To be consistent with previous AHRI analyses, we included men aged 15–54 years and women aged 15–49 years that tested for HIV between 2005 and 2018. Participants had to be repeat-testers, with a first HIV-negative test result followed by at least one valid HIV test result. We identified all repeat-testers whose infection event was censored between the latest HIV-negative and earliest HIV-positive test dates. For these repeat-testers, we imputed the infection dates in two stages.

For the first stage, we used a G-transformation model to quantify the effects of the time-dependent covariates on the interval censored infection events. The G-transformation model is described in greater detail by Zeng et al. elsewhere [11, 14]. Let *T* denote the infection time and let *Z*(·) denote a d-vector of covariates. Under the semiparametric transformation model, the cumulative hazard function for *T* conditional on *Z*(·) can be written as:

1 $$ \Lambda(t; Z) = G \left(\int_{0}^{t} e^{\beta^{\intercal} Z(s)} d\Lambda(s) \right)  $$

where *β* is a d-vector of regression parameters, *Λ*(·) is an unknown increasing function, and *G*(·) is a strictly increasing transformation function [11]. For this analysis, we selected *G*(*x*)=*log*(1+*x*), which gives the proportional odds model. The G-transformation model does not require imputed infection times as input or return imputed infection times as output.

Based on previous work from the AHRI surveillance area, we selected covariates that had a well-established association with the risk of HIV acquisition. We grouped these as follows: *Individual covariates*: age (5 year categories from 15 to 40 years, and >40 years) and circumcision status (circumcised or not) [9, 15, 16]. Only men were asked to self-report their circumcision status in the HIV surveys. *Behavioral covariates*: marital status (single vs. married), condom use (always vs. sometimes), and cumulative time spent outside the surveillance area (low, moderate, high) [12, 15–17, 19–21]. *Structural covariates*: area of residence (urban, peri-urban, rural), household socio-economic status (low, middle, high), and opposite-sex HIV prevalence [8, 15, 21–23]. The community HIV prevalence is a geospatial measure that we constructed using methods described elsewhere [8].

For comparison with the multivariate models, we also ran a model with no covariates and undertook univariate analyses for each covariate. All covariates were treated as time-dependent. Because of the large differential in HIV acquisition risk between men and women, we stratified the analyses by sex [15, 23–25].

For the second stage, we used the G-transformation model results to derive the participant’s unique cumulative distribution function and sample multiple infection dates conditional on his or her covariate values [10]. For the *i*^+^th HIV-positive repeat-tester, the unique cumulative distribution function is given by:

2 $$ \hat{F}_{i^{+}}(t; Z_{i^{+}},\hat{\beta}) = 1 - e^{-G \left(\sum_{\{j:u_{j} \leq t\}} e^{\hat{\beta}^{\intercal} Z_{i^{+}}(u_{j})} \left[\hat{\Lambda}(u_{j}) - \hat{\Lambda}(u_{j-1}) \right] \right)},  $$

which is also a right-continuous, non-decreasing step function having knots at $u_{1} \leq u_{2} \leq \dots \leq u_{m}$ with *u*_1_≥0 and *u*_*m*_≤*U* [10]. If we define $\hat {F}_{i^{+}}(t;Z_{i^{+}},\hat {\beta })= \hat {F}_{i^{+}}(u_{j};Z_{i^{+} },\hat {\beta })$ for any *t*∈(*u*_*j*_,*u*_*j*+1_], then the probability of sampling an infection date within the *i*^+^th censoring interval is:

3 $$ Pr(T_{i^{+}}= u_{j} | \hat{\beta}) = \hat{F}_{i^{+}}(u_{j};Z_{i^{+} },\hat{\beta}) - \hat{F}_{i^{+}}(u_{j-1};Z_{i^{+} },\hat{\beta}).  $$

We right censored the data at the imputed date (or at the latest HIV-negative date for all uninfected repeat-testers), and calculated the annual incidence rate per 100 person-years. To quantify the uncertainty of our approach, we generated 300 imputed datasets and obtained the means of the annual estimates and their standard errors using Rubin’s rules [26]. For the single random-point method, we sampled the infection dates from a uniform distribution, as described in detail elsewhere [6], and calculated the incidence rates using the same multiple imputation strategy.

All analyses were performed with R statistical software, version 3.6.3.

## Results

Participation rates for the HIV incidence cohort are shown in Table S1. On average, 71% (range: 64–77%) of the eligible HIV-negative participants had a repeat HIV test, entered the incidence cohort, and contributed person-time to the analysis between 2005 and 2018. Figure S1 shows that there were no significant differences between the eligible HIV-negative participants that did and did not enter the incidence cohort. Of the 20,011 repeat-testers that entered the cohort, 11,412 were women aged 15–49 years and 8,599 were men aged 15–54 years. We observed 2,603 infections per 58,769 person-years of follow-up among women, with an overall incidence of 4.43 infections per 100 person-years. Among men, we observed 845 infections per 41,178 person-years of follow-up with an overall incidence of 2.05 infections per 100 person-years. The median length of the censoring interval was 3 years.

The G-transformation model results for the individual, behavioral, and structural level covariates are shown in Table 1 (univariate) and Table 2 (multivariate). For the individual covariates in Table 2, the risk of HIV acquisition was highest among men in the 30–34 year age group (HR=6.175, 95% CI=[5.094, 7.486]) and among women in the 20–24 year age group (HR=1.382, 95% CI=[1.250, 1.528]), when compared with 15–19 year olds. Male circumcision was also associated with a reduced risk of HIV acquisition among men (HR=0.729, 95% CI=[0.558, 0.953]), holding age constant.

**Table 1:** Univariate results showing the G-transformation hazard ratios for the individual, behavioral, and structural level covariates of HIV acquisition

	Men			Women		
	HR	95% CI	*P*-value	HR	95% CI	*P*-value
*Individual Variables*						
Age (vs. 15–19 years):						
20–24	3.795	(3.211, 4.485)	<0.001	1.382	(1.250, 1.528)	<0.001
25–29	5.707	(4.888, 6.662)	<0.001	1.161	(1.033, 1.305)	0.012
30–34	6.367	(5.302, 7.647)	<0.001	0.990	(0.846, 1.158)	0.896
35–39	5.085	(3.820, 6.769)	<0.001	0.374	(0.296, 0.472)	<0.001
40+	2.627	(2.155, 3.203)	<0.001	0.242	(0.202, 0.289)	<0.001
Circumcised (vs. Uncircumcised)	0.694	(0.548, 0.879)	0.002			
*Behavioral Variables*						
Married (vs. Single)	0.636	(0.504, 0.803)	<0.001	0.327	(0.284, 0.378)	<0.001
Condom use: Sometimes (vs. Always)	1.362	(1.172, 1.582)	<0.001	1.465	(1.347, 1.592)	<0.001
Cum. Outmigration (vs. Low): ^*†*^						
Moderate	0.914	(0.770, 1.086)	0.306	1.594	(1.457, 1.744)	<0.001
High	1.186	(1.001, 1.404)	0.048	1.435	(1.298, 1.588)	<0.001
*Structural Variables*						
Household SES (vs. Lower tertile)						
Middle tertile	1.225	(1.051, 1.428)	0.009	1.080	(0.977, 1.194)	0.133
Upper tertile	1.135	(1.001, 1.287)	0.049	1.093	(1.003, 1.191)	0.044
Area of residence (vs. Urban):						
Rural	0.656	(0.562, 0.767)	<0.001	0.774	(0.716, 0.838)	<0.001
Peri-urban	1.210	(0.910, 1.609)	0.190	1.007	(0.781, 1.299)	0.956
HIV Prevalence (vs. Low): ^*‡*^						
Moderate	1.745	(1.371, 2.222)	<0.001	1.365	(1.239, 1.505)	<0.001
High	2.337	(1.964, 2.781)	<0.001	1.593	(1.440, 1.761)	<0.001

Hazard ratio (HR), Standard error (SE), Confidence interval (CI). Hazard ratios were obtained with the G-transformation model for interval censored data

*†*Low, moderate, and high defined as <5%, 5–20%, and >20% cumulative time spent outside of the study area for men and women

*‡*Opposite-sex HIV prevalence of the participant’s surrounding community. Low, moderate, and high prevalence defined as <10%, 10–20%, and >20% for men and <15%, 15–25%, >25% for women, respectively. Different categories used because of the large difference in HIV prevalence among men and women

**Table 2:** Multivariate results showing the G-transformation hazard ratios for the individual, behavioral, and structural level covariates of HIV acquisition

	Men			Women		
	HR	95% CI	P-value	HR	95% CI	*P*-value
*Individual Variables*						
Age (vs. 15–19 years):						
20–24	3.743	(3.131, 4.476)	<0.001	1.382	(1.250, 1.528)	<0.001
25–29	5.554	(4.714, 6.544)	<0.001	1.161	(1.033, 1.305)	0.012
30–34	6.175	(5.094, 7.486)	<0.001	0.990	(0.846, 1.158)	0.896
35–39	4.948	(3.704, 6.611)	<0.001	0.374	(0.296, 0.472)	<0.001
40+	2.523	(2.051, 3.104)	<0.001	0.242	(0.202, 0.289)	<0.001
Circumcised (vs. Uncircumcised)	0.729	(0.558, 0.953)	0.021			
*Behavioral Variables*						
Married (vs. Single)	0.619	(0.466, 0.823)	<0.001	0.396	(0.343, 0.459)	<0.001
Condom use: Sometimes (vs. Always)	0.973	(0.830, 1.140)	0.734	0.628	(0.574, 0.687)	<0.001
Cum. Outmigration (vs. Low): ^*†*^						
Moderate	0.883	(0.738, 1.056)	0.172	1.304	(1.186, 1.433)	<0.001
High	1.163	(0.976, 1.387)	0.091	1.197	(1.077, 1.330)	<0.001
*Structural Variables*						
Household SES (vs. Lower tertile)						
Middle tertile	0.847	(0.678, 1.060)	0.146	0.955	(0.856, 1.066)	0.412
Upper tertile	0.933	(0.776, 1.121)	0.459	0.999	(0.904, 1.104)	0.987
HIV Prevalence (vs. Low): ^*‡*^						
Moderate	0.940	(0.566, 1.560)	0.811	1.361	(1.205, 1.536)	<0.001
High	1.325	(0.885, 1.983)	0.171	1.601	(1.413, 1.815)	<0.001

Hazard ratio (HR), Standard error (SE), Confidence interval (CI). Hazard ratios were obtained with the G-transformation model for interval censored data

*†*Low, moderate, and high defined as <5%, 5–20%, and >20% cumulative time spent outside of the study area for men and women

*‡*Opposite-sex HIV prevalence of the participant’s surrounding community. Low, moderate, and high prevalence defined as <10%, 10–20%, and >20% for men and <15%, 15–25%, >25% for women, respectively. Different categories used because of the large difference in HIV prevalence among men and women

For the behavioral covariates in Table 2, being married was protective against HIV acquisition among men (HR=0.619, 95% CI=[0.466, 0.823]) and women (HR=0.396, 95% CI=[0.343, 0.459]) when compared with being single, holding all else constant. For self-reported condom use, men (HR=0.973, 95% CI=[0.830, 1.140]) and women (HR=0.628, 95% CI=[0.574, 0.687]) had a lower risk of HIV acquisition when compared with using condoms sometimes. Compared with low cumulative out-migration, a high (HR=1.163, 95% CI=[0.976, 1.387]) amount of cumulative time spent outside the study area was associated with a higher risk of HIV acquisition among men. Among women, moderate (HR=1.304, 95% CI=[1.186, 1.433]) and high (HR=1.197, 95% CI=[1.077, 1.330]) cumulative out-migration was associated with a higher risk of HIV acquisition.

For the structural covariates, living in a household with a higher socio-economic status increased the HIV acquisition risk for men and women, as shown in the univariate models in Table 1. In the multivariate model in Table 2, household socio-economic status was not significant. Because area of residence and HIV prevalence were highly correlated, we dropped the former variable from the multivariate model. Living in a community with a high HIV prevalence was associated with a higher HIV acquisition risk among men (HR=1.325, 95% CI=[0.885, 1.983]) and women (HR=1.601, 95% CI=[1.413, 1.815]), when compared with living in a community with low HIV prevalence.

**Fig. 1:**
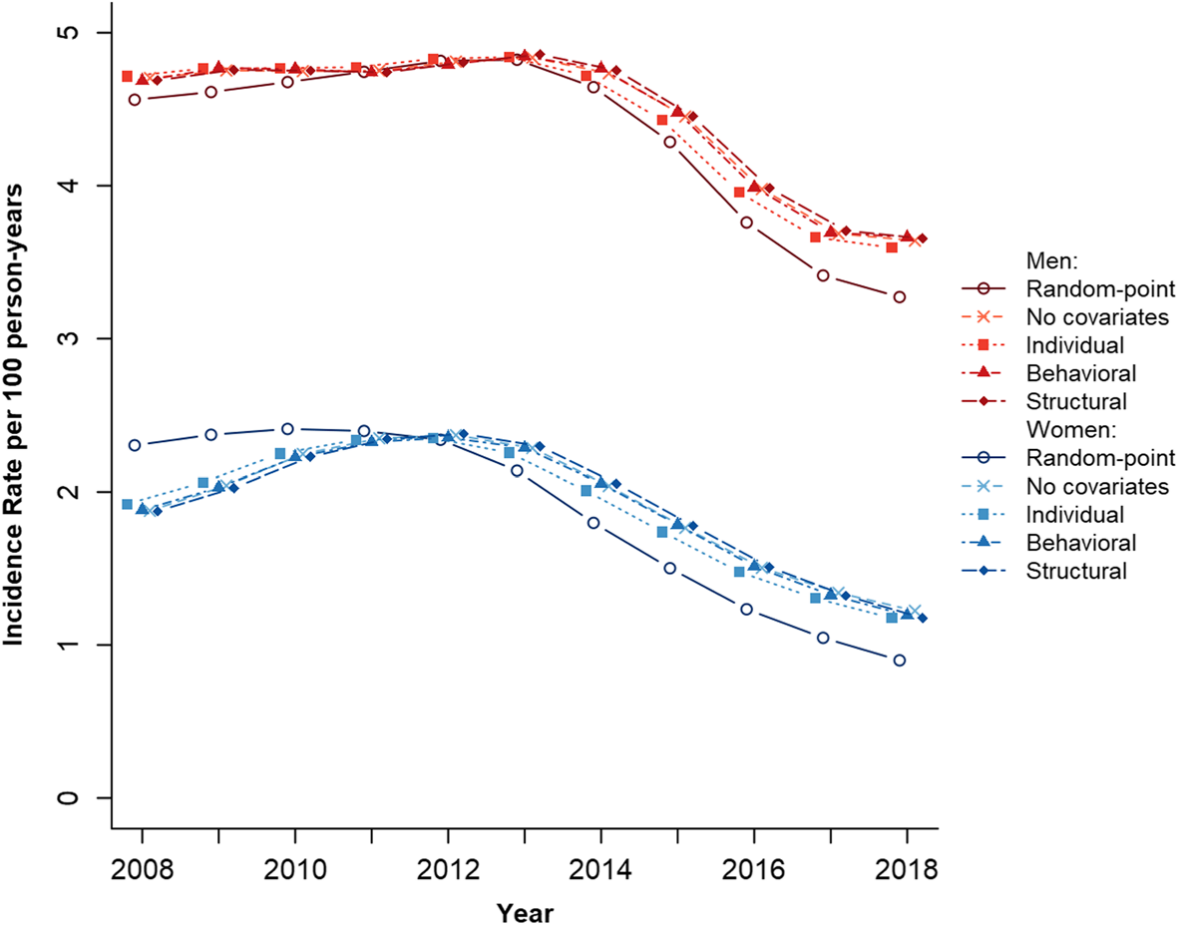
Shows the male and female HIV incidence rates computed from the single random-point method and four G-imputation models with 1) no covariates, 2) individual-level covariates (age, circumcision status [for men only]), 3) behavioral-level covariates (marital status, condom use, cumulative out-migration), and 4) structural-level covariates (household socio-economic status, HIV prevalence)

In Fig. 1, we compare the HIV incidence rates from the single random-point method, a G-imputation model with no covariates, and the G-imputation models with the individual, behavioral, and structural level covariates. The G-imputation estimates and their 95% confidence intervals are presented in Table S2 of the Supplement. Across all models, the incidence rate was higher in women than men and incidence declines occurred earlier in men than women. The incidence rates did not differ markedly across the individual, behavioral, and structural models, with the individual model showing the largest declines in incidence. For the individual model, the female incidence declined by 25% from 5.0 to 3.7 infections per 100 person-years between 2014 and 2018. Conditional on age and circumcision status, the incidence rate among men fell from 2.1 to 1.1 infections per 100 person-years between 2014 and 2018—a decline of 49%. Relative to the G-imputation models, the single random-point estimates show a slightly larger decline in incidence from 2011 onward for men and from 2013 onward for women.

## Discussion

Accurate incidence rate estimates are critically needed to evaluate the effectiveness of HIV prevention strategies in sub-Saharan Africa. One way to measure the population-wide incidence is to repeatedly test a large cohort of uninfected participants until they are HIV-positive [2]. However, due to periodic testing and missed test dates, we rarely (if ever) observe the exact infection date, which is known only to occur at some time-point between the latest HIV-negative and earliest HIV-positive test dates (the censoring interval). We have previously shown that ad hoc assumptions about the infection date—for example, imputation of the event at the mid-point of the censoring interval—can lead to artefactual trends in the HIV incidence rate [6]. Recently, we demonstrated a novel statistical approach, called G-imputation, to infer the infection date conditional on covariate information associated with the risk of HIV acquisition [10]. In a simulation analysis, we showed that incidence rate estimates from the G-imputation method were close to the true estimates, and were more accurate than estimates from the mid-point method and single random-point method using multiple imputation [10]. Here, we used the G-imputation approach, with data collected from one of the world’s largest and longest running HIV cohorts, to accurately estimate trends in the population-wide incidence of infection.

The first stage of our imputation approach required the use of a G-transformation model to estimate the effects of the time-dependent covariates on the interval censored infection dates [11, 14]. Our results show that women aged 15–24 years and men aged 25–34 years had the highest risk of HIV acquisition. This age-specific risk can be attributed to the age structure of sexual partnerships in the surveillance area. Men aged 25–34 years are most likely to report recent sexual activity with women aged 25–34 years, which is the period of peak female HIV prevalence. These men, who are more likely to be recently infected, will have a higher risk of transmitting HIV to their younger female partners (aged 15–24 years), who then age into the group of similar-aged male partners [25, 27]. One factor that could reduce the risk of HIV acquisition among men, and therefore disrupt the cyclical structure of onward HIV transmission, is voluntary medical male circumcision (VMMC). Male circumcision is strongly associated with a reduced risk of HIV acquisition among men in sub-Saharan African settings [17, 28]. Our results confirm that male circumcision was a significant protective factor against HIV acquisition. This finding is broadly consistent with the introduction of a local VMMC program in 2009, where 33% of all men reporting being circumcised by 2016 [17]. Because age and circumcision status are strongly associated with the risk of HIV acquisition, we used this covariate to infer the date of infection.

We report that more cumulative time spent outside the study area increased both the male and female risk of HIV acquisition, a result that is supported by previous research [12, 16, 19, 20, 29]. For example, one of these studies showed that the HIV acquisition risk increased by 50% when men spent 44% and women 90% of their time away from their household residence [12]. Mobility patterns in the study area are largely shaped by the legacy of apartheid-era policies, which sought to regulate the rural supply of African labourers into urban centres and prevent their spouses or families from joining them [30]. Presently, the lack of local employment opportunities and ongoing cyclical migration has resulted in the extended absence of men from the rural family home, leading to marital instability [31, 32]. Research has shown that resident participants who spend fewer nights in the study area are more likely to have higher levels of risky sexual behavior and less likely to access HIV prevention services [33–35]. Thus, the strong association between migration and HIV acquisition risk motivated the inclusion of this covariate data in our imputation strategy.

Our results show that higher prevalence of HIV in the surrounding community was significantly associated with increased HIV acquisition risk, as we have demonstrated elsewhere [8, 15]. Higher HIV prevalence means that uninfected residents will have an increased probability of meeting, having sexual contact, and acquiring HIV from an infected resident. In the AHRI surveillance area, HIV prevalence is highest (>35%) in the informal urban settlements near the N2 highway while the more inaccessible rural areas near the western boundary have the lowest HIV prevalence (<10%) [22]. For this reason, the urban/rural status of a community is strongly correlated with a measure of its HIV prevalence. Our results show, for example, that living in an urban area was associated with a higher risk of HIV acquisition when compared with living in rural areas, which is consistent with previous research from the surveillance area [16, 21]. We therefore included HIV prevalence as covariate information in our analysis, as we would expect those living in high prevalence (urban) areas to have shorter times to infection than participants living in low prevalence (rural) areas.

In the second stage, we used the G-transformation model results and the participant’s covariate values to infer the HIV infection date. We used a model with no covariates, individual-level covariates only, behavioral-level covariates only, and structural-level covariates only. The four models show significant declines in the HIV incidence rates for men and women. For example, conditional on age, the female incidence rate declined by 25%, from 5.0 to 3.7 infections per 100 person-years between 2014 and 2018 (see Table S2). During this period, the male incidence (conditional on age and circumcision status) declined from 2.1 to 1.1 infections per 100 person-years between 2014 and 2018—a reduction of 49%. As we describe elsewhere [24], the larger decline in male incidence is likely due to the introduction of a VMMC programme in 2009, the scale-up of HIV testing and counseling services in 2010, and female ART coverage surpassing 35% in 2012. Among women, declines in HIV incidence began once men reached similar levels of ART coverage [17].

In this study, we confirm that the HIV incidence rate in the AHRI surveillance area has declined markedly since 2012. Previously, we used the single random-point method and standard multiple imputation procedures to demonstrate real declines in the incidence of HIV infection [6, 17]. These declines among men and women indicate gradual progress toward HIV epidemic control [36, 37]. However, HIV incidence remains high, particularly among women. We have previously argued that the high female HIV incidence reflects a differential in the uptake of treatment among men, who have lower ART coverage and higher levels of detectable viremia [17, 24, 38, 39]. Research from the ongoing HIV incidence cohort, together with the findings from the ANRS Treatment-as-Prevention trial in the same community [40], suggests that innovative health systems approaches will be needed to get people in early stages of HIV infection onto treatment [37].

Our HIV incidence results are broadly consistent with other studies from South Africa and sub-Saharan Africa. In a community-based cohort study from a (different) rural area in KwaZulu-Natal, the HIV incidence rate among young women (aged 15–19 years) declined from 4.6 to 2.7 per 100 person-years between 2014 and 2018. However, declines among men and women in the older age groups were marginal or remained unchanged [41]. Similar to our findings, representative and cross-sectional studies from eSwatini (prevoiusly Swaziland) and South Africa have reported reductions in both the male and female HIV incidence rate between 2011 and 2017 [42, 43]. Two other population-based cohorts from Uganda and Kenya have also reported reductions of between 42–50% in the overall incidence of HIV between 2012 and 2016 [44, 45]. Together, these studies show that a combination of treatment preventions has been successful in reducing HIV incidence at the population level, but that greater coverage of these strategies is needed to reach key milestones by 2030 [46].

## Conclusion

In conclusion, we used the G-imputation approach with covariate data collected from one of the world’s largest and longest running HIV cohorts to estimate trends in the population-wide incidence of infection. Our results show that the female incidence rate declined by 25%, from 5.0 to 3.7 infections per 100 person-years between 2014 and 2018. During the same period, the HIV incidence rate among men declined from 2.1 to 1.1 infections per 100 person-years—a reduction of 49%. Our G-imputation results provide further evidence to support the observation that the HIV incidence rate among men and women has been declining over time. This work contributes to ongoing efforts to improve the accuracy of incidence rate estimates following the scale-up of HIV prevention services in sub-Saharan Africa.

## Supplementary information

**Additional file 1** Supplementary Figure and Tables.

## Data Availability

All relevant data supporting the key findings of this study are available within the article and its Supplementary Information files. The datasets used for the analysis presented in this study are available from the Africa Health Research Institute (AHRI) data repository: https://data.africacentre.ac.za/index.php/auth/login/?destination=. To access the licensed datasets, the applicant must agree to the terms and conditions of use by completing an Application for Access to a Licensed Dataset. This request will be reviewed by the AHRI Data Release Committee, who may decide to approve the request, to deny access to the data, or to request additional information from the applicant. The code for the G-imputation approach is available in the R *ahri* package at https://github.com/vando026/ahri.

## Abbreviations

HIV: Human immunodeficiency virus
AIDS: Acquired immune deficiency syndrome
AHRI: Africa Health Research Institute
ANRS: Agence nationale de recherches sur le sida et les hépatites virales
ART: antiretroviral therapy
CI: confidence interval
ELISA: Enzyme-linked immunosorbent assay
UNAIDS: Joint United Nations Programme on HIV/ Acquired Immune Deficiency Syndrome
VMMC: voluntary medical male circumcision
WHO: World Health Organization
