# Supplementary material for: HIV incidence declines in a rural South African population: a G-imputation approach for inference
Source: BMC Public Health. 2020 Aug 6;20:1205. doi: 10.1186/s12889-020-09193-4 (PMC7409400; doi:10.1186/s12889-020-09193-4)
Supplement: Supplementary file 1 — Additional file 1 Supplementary Figure and Tables. [file 12889_2020_9193_MOESM1_ESM.pdf]

SUPPLEMENT: HIV incidence declines in a rural South African population: a G-imputation approach for inference

Alain Vandormael[1,2,3*], Diego Cuadros[4], Adrian Dobra[5], Till Bärnighausen[1,2,6], and Frank Tanser[1,7,8,9]

[1]Africa Health Research Institute (AHRI), Durban, South Africa.
[2]Heidelberg Institute of Global Health, University of Heidelberg, Heidelberg, Germany.
[3]KwaZulu-Natal Research Innovation and Sequencing Platform (KRISP), University of KwaZulu-Natal (UKZN), Durban, South Africa.
[4]Department of Geography and Geographic Information Science, University of Cincinatti, Cincinatti, USA
[5]Department of Statistics, Center for Statistics and the Social Sciences, and Center for Studies in Demography and Ecology, University of Washington, Seattle, USA
[6]Department of Global Health and Population, Harvard T.H. Chan School of Public Health, Boston, USA.
[7]Lincoln International Institute for Rural Health, University of Lincoln, Lincoln, United Kingdom.
[8]School of Nursing and Public Health, UKZN, Durban, South Africa.
[9]Centre for the AIDS Programme of Research in South Africa (CAPRISA), Durban, South Africa.
[*]Corresponding author: alain.vandormael@uni-heidelberg.de

Supplementary Figures

Figure S1: Compares the basic demographic characteristics of repeat-testers that entered the HIV cohort (labeled as HIV cohort) with eligible HIV-negative participants that did not enter the HIV cohort (labeled as HIV-negatives). The comparison between both groups is for the percentage that were women, the mean age (in years) by sex, and the mean number of in-migration and out-migration events irrespective of serostatus. The figure show that the basic demographic characteristics not markedly differ between the two groups.

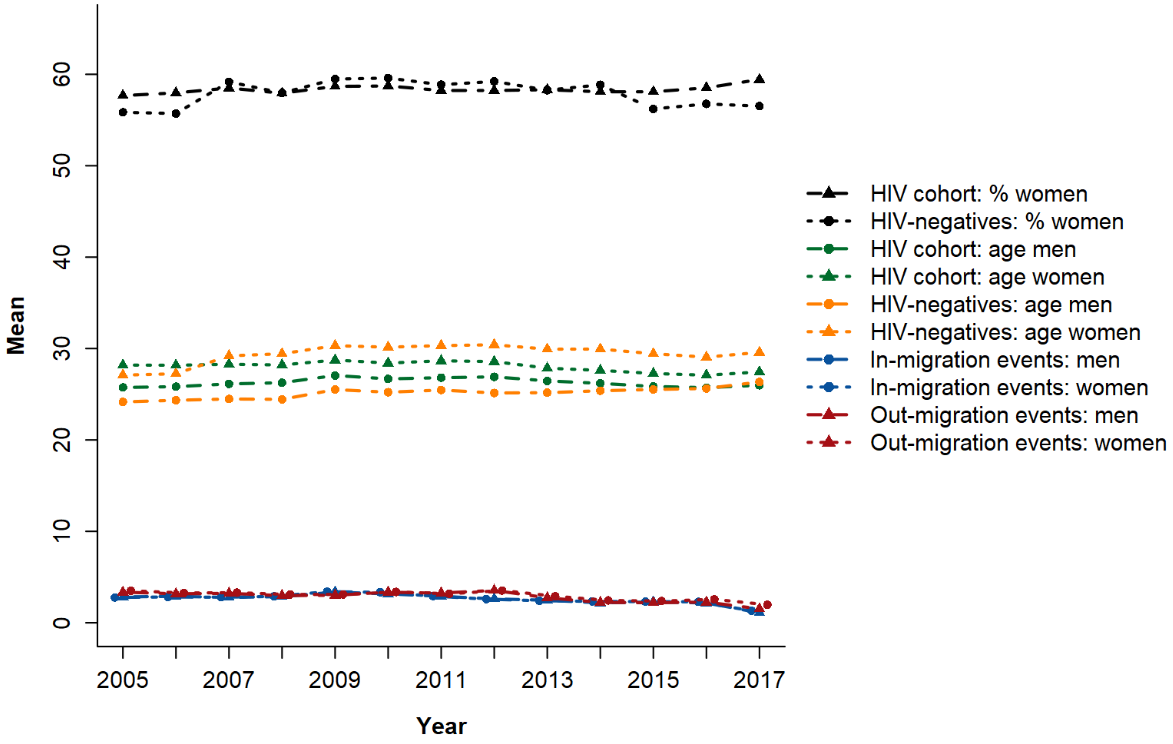

Supplementary Tables

Table S1: Participation in the HIV incidence cohort.

	HIV$^-$ Eligible[1]	Repeat-testers[2]	
Year	no.	no.	(%)
2005	13,568	10,043	(74.0)
2006	15,854	11,629	(73.3)
2007	17,819	13,006	(73.0)
2008	19,622	14,271	(72.7)
2009	20,671	14,924	(72.2)
2010	23,021	16,476	(71.6)
2011	24,547	17,351	(70.7)
2012	26,000	18,308	(70.4)
2013	27,962	19,592	(70.1)
2014	29,464	20,641	(70.0)
2015	31,551	21,941	(69.5)
2016	33,870	23,052	(68.1)
2017	35,254	23,554	(66.8)
2018	36,840	23,554	(63.9)

[1]Shows the number of HIV-negative participants that were eligible for entry into the HIV incidence cohort at the household visit date. [2]Shows the number and percentage of eligible HIV-negative participants that had a repeat-test, entered into the HIV cohort, and contributed person-time to the analysis.

Table S2: Incidence rates and 95% confidence intervals computed from four G-imputation models with no covariates, individual-level, behavioral-level, and structural-level covariates.

	**No covariates**		**Individual**		**Behavioral**		**Structural**	
	**Rate**	**(95% CI)**	**Rate**	**(95% CI)**	**Rate**	**(95% CI)**	**Rate**	**(95% CI)**
Males								
2008	1.71	(1.13, 2.30)	1.75	(1.15, 2.36)	1.73	(1.13, 2.33)	1.71	(1.13, 2.29)
2009	2.04	(1.38, 2.70)	2.05	(1.37, 2.72)	2.00	(1.36, 2.63)	2.02	(1.35, 2.70)
2010	2.39	(1.69, 3.09)	2.40	(1.72, 3.09)	2.39	(1.67, 3.11)	2.35	(1.65, 3.05)
2011	2.32	(1.65, 3.00)	2.29	(1.56, 3.02)	2.28	(1.57, 2.99)	2.32	(1.63, 3.00)
2012	2.42	(1.67, 3.17)	2.43	(1.67, 3.19)	2.40	(1.66, 3.15)	2.45	(1.72, 3.19)
2013	2.41	(1.64, 3.18)	2.35	(1.64, 3.05)	2.42	(1.68, 3.17)	2.40	(1.67, 3.14)
2014	2.11	(1.43, 2.78)	2.09	(1.41, 2.78)	2.12	(1.44, 2.80)	2.14	(1.47, 2.82)
2015	1.68	(1.09, 2.27)	1.64	(1.04, 2.24)	1.71	(1.12, 2.30)	1.67	(1.06, 2.28)
2016	1.49	(0.90, 2.07)	1.49	(0.95, 2.04)	1.52	(0.91, 2.14)	1.53	(0.93, 2.14)
2017	1.30	(0.67, 1.94)	1.25	(0.65, 1.85)	1.26	(0.64, 1.88)	1.27	(0.66, 1.87)
2018	1.13	(0.27, 1.99)	1.07	(0.16, 1.98)	1.09	(0.22, 1.97)	1.06	(0.19, 1.93)
Females								
2008	4.67	(3.83, 5.51)	4.72	(3.92, 5.52)	4.67	(3.89, 5.45)	4.63	(3.85, 5.41)
2009	4.79	(3.99, 5.59)	4.76	(3.93, 5.60)	4.84	(3.98, 5.70)	4.85	(4.02, 5.69)
2010	4.83	(3.99, 5.67)	4.88	(4.05, 5.71)	4.87	(4.04, 5.70)	4.82	(3.96, 5.67)
2011	4.53	(3.71, 5.34)	4.54	(3.71, 5.37)	4.48	(3.63, 5.34)	4.52	(3.70, 5.34)
2012	5.01	(4.10, 5.93)	5.04	(4.11, 5.96)	4.98	(4.09, 5.86)	4.96	(4.07, 5.85)
2013	4.75	(3.93, 5.57)	4.76	(3.88, 5.64)	4.75	(3.91, 5.59)	4.81	(3.93, 5.69)
2014	4.98	(4.16, 5.79)	4.95	(4.10, 5.80)	5.04	(4.13, 5.94)	5.03	(4.18, 5.88)
2015	4.55	(3.73, 5.36)	4.52	(3.66, 5.38)	4.58	(3.77, 5.40)	4.50	(3.69, 5.31)
2016	3.97	(3.19, 4.74)	3.93	(3.16, 4.71)	3.97	(3.15, 4.79)	3.96	(3.20, 4.73)
2017	3.25	(2.45, 4.04)	3.27	(2.51, 4.03)	3.23	(2.43, 4.02)	3.32	(2.54, 4.09)
2018	3.82	(2.67, 4.97)	3.73	(2.55, 4.91)	3.87	(2.64, 5.10)	3.81	(2.60, 5.01)
